# The relations of dosimetric parameters with long‐term outcomes and late toxicities in advanced T‐stage nasopharyngeal carcinoma with IMRT

**DOI:** 10.1002/hed.25986

**Published:** 2019-10-24

**Authors:** Xiaoxia Gou, Baofeng Duan, Huashan Shi, Lei Qin, Jianghong Xiao, Nianyong Chen

**Affiliations:** ^1^ Department of Head and Neck Oncology and Department of Radiation Oncology, Cancer Center and State Key Laboratory of Biotherapy, West China Hospital Sichuan University Chengdu China; ^2^ Department of Head and Neck Oncology Affiliated Hospital of Zunyi Medical University Zunyi China

**Keywords:** clinical outcomes, dosimetry, intensity‐modulated radiation therapy, nasopharyngeal carcinoma, organ at risk

## Abstract

**Background:**

Balancing the dose requirements between targets and normal tissue is a challenge in radiation of nasopharyngeal carcinoma (NPC). The purpose of this study is to evaluate the dosimetric parameters and clinical outcomes in NPC.

**Methods:**

We presented a retrospective review of patients with T3‐4 NPC treated by intensity‐modulated radiation therapy (IMRT). Patient characteristics, dosimetric parameters, and the follow‐up data for survival and late toxicities were analyzed.

**Results:**

The 5‐year overall survival, local relapse‐free survival, and distant metastasis‐free survival were 83.0%, 90.1%, and 82.4%, respectively. Multivariate analysis revealed that the volume of involved lymph node was an independent prognostic factor. The volume of primary tumor and the maximal dose were significant factors affecting temporal lobe injury.

**Conclusions:**

IMRT provided satisfactory local control for advanced T‐stage NPC, with acceptable late toxicities. The dose constraint criteria of selected critical structures can be appropriately loosen.

## 1 INTRODUCTION

Nasopharyngeal carcinoma (NPC) is a cancer arising from the nasopharyngeal epithelium, with high incidence in Southern China and Southeast Asia. Radiotherapy (RT) is the primary and only curative treatment for nonmetastatic NPC.^1^, ^2^ Concurrent chemoradiotherapy is recommended as a standard treatment for locoregionally advanced NPC.^3^

Intensity‐modulated radiation therapy (IMRT), which delivers a high dose to the tumor while a low‐dose spares normal tissues, is considered an effective development in radiation therapy. However, for patients with advanced T‐stage NPC, especially those with T4 stage, a common issue encountered in planning IMRT is that the space between the primary tumor and neurological structures is extremely narrow.^4^ Thus, RT in patients with T3‐4 NPC is technically challenging, particularly in patients with intracranial extension involving structures such as the brainstem, spinal cord, temporal lobes, optic chiasm, and optic nerves. To achieve satisfactory dose coverage to the target volumes, a certain portion of the organs at risk (OARs) is more likely to be involved in the radiation fields and receive an unavoidable high dose of irradiation. Due to the large tumor volume and advanced disease, patients experience late radiation‐induced complications, including temporal lobe injury (TLI), ototoxicity, xerostomia, neck fibrosis, and transverse myelitis. For patients with advanced T‐stage NPC, we often face the clinical issues of target coverage and OARs protection in IMRT planning. In fact, there are different choices for balancing clinical needs. Normally, tumor target areas adjacent to the critical OARs cannot reach the prescription dose requirements. It remains unknown whether an insufficient dose to the tumor target reduces tumor control. However, if the dose requirement of the tumor target volume is met, the dose received by the critical OARs adjacent to the tumor usually exceeds the dose limit requirement of the critical OARs. In that case, whether the excess of this limited dose leads to serious complications of the OARs remains unknown.

In this study, we analyzed the actual dose of NPC treated with IMRT. We also addressed whether the excess of some doses received by the OARs had serious complications. Therefore, we assessed clinical outcomes and late radiation‐induced toxicities in advanced T‐stage NPC and investigated dosimetric predictors for patients who develop TLI after IMRT.

## 2 PATIENTS AND METHODS

### 2.1 Patient characteristics

Between January 2010 and December 2012, 200 newly diagnosed, biopsy‐proven patients with stage T3‐4 nonmetastatic NPC were treated with IMRT at the West China Hospital of Sichuan University. All patients were pathologically diagnosed by biopsy and tested by nasopharyngoscopy, nasopharyngeal and neck MRI, chest CT, abdominal ultrasonography or CT, whole‐body bone scan using single photon emission CT, and dental assessment. Patients were staged using the seventh edition of the staging system jointly used by the American Joint Committee of Cancer and International Union Against Cancer.^5^ The clinical characteristics of the patients are summarized in Table 1. A total of 166 patients underwent MRI for TLI evaluation during the follow‐up period. This study was approved by the ethics committee of the hospital, and informed consent was waived.

**Table 1:** Patient characteristics and treatment factors (n = 200)

Characteristics	No. of patients (%)
Age (years)	
Median	52
Range	19‐73
Sex	
Male	147 (73.5)
Female	53 (26.5)
T classification	
T3	80 (40.0)
T4	120 (60.0)
N classification	
N0	22 (11.0)
N1	55 (27.5)
N2	107 (53.5)
N3	16 (8.0)
Stage	
III	74 (37.0)
IVA/IVB	126 (63.0)
Prescription dose (Gy)	
70	95 (47.5)
74	105 (52.5)
Chemotherapy	
≤4 cycles	102 (51.0)
>4 cycles	98 (49.0)
Targeted therapy	
Yes	34 (17.0)
No	210 (83.0)

### 2.2 Radiotherapy

Inverse IMRT planning was performed with the Pinnacle system version 9.0. Target volumes were delineated according to our institutional treatment protocol, the International Commission on Radiation Units and Measurements reports 50, 62, and 83. Gross tumor volume (GTV) including the primary nasopharyngeal tumor (GTVnx) and involved lymph nodes (GTVnd) was delineated based on simulation CT coregistered with diagnostic MRI. Volume of GTVnx included GTV of nasopharynx, and volume of GTVnd included the sum volume of all involved lymph nodes. Clinical target volumes (CTVs) were delineated on the basis of tumor invasion as follows: CTV1, high‐risk regions including GTV plus 5‐10 mm, and entire nasopharynx. CTV2, low‐risk regions containing the CTV1 plus a 5‐10 mm margin, including the parapharyngeal space, skull base, pterygoid fossae, clivus, inferior sphenoid sinus, posterior third of the nasal cavity, maxillary sinuses, retropharyngeal lymph nodal regions, and the elective nodal regions from level IB to level V. Planning target volumes, termed PGTVnx, PGTVnd, PCTV1, and PCTV2, were constructed by expanding the GTVnx, GTVnd, CTV1, and CTV2, respectively, by 3 mm. OARs, including the brainstem, spinal cord, optic chiasm, optic nerves, lens, temporal lobes, pituitary, larynx, mandible, temporomandibular joint, inner ear, and parotid glands, were carefully outlined.

The dose prescribed was 70‐74 Gy to the PGTVnx, 70 Gy to the PGTVnd, 60 Gy to the PCTV1, and 50‐56 Gy to the PCTV2 in 33 fractions. All patients received one fraction daily, 5 days per week. The plan consisted of multileaf collimator segments of 6 MV isocentric, coplanar beams arranged in seven almost equally spaced beam angles. The dose received by each normal tissue constraint followed the protocol of the Radiation Therapy Oncology Group (RTOG) 0225 and RTOG 0615.^6^, ^7^ The dosimetric parameters were obtained from the dose‐volume histogram and included the volume (V) of the PGTVnx, PGTVnd, PCTV1 and PCTV2; the near‐maximum dose (D2); the near‐minimum dose (D98); the median dose (D50); the dose to 95% of the target volume (D95); the conformity index^8^; and the homogeneity index.^9^

### 2.3 Chemotherapy

Of all the patients with stage III or IVA‐B NPC, only 1.5% (3/200) were treated with RT alone due to personal reasons or physical factors. A total of 98.5% (197/200) of patients received chemotherapy, including induction chemotherapy and concomitant chemotherapy +/− adjuvant chemotherapy. Induction chemotherapy or adjuvant chemotherapy consisted of cisplatin with 5‐fluorouracil (PF), docetaxel with cisplatin (TP), or a triplet of docetaxel, cisplatin, and 5‐fluorouracil (TPF) every 3 weeks for two to four cycles before or after RT. Concurrent chemotherapy consisted of cisplatin (80‐100 mg/m^2^) given on days 1, 22, and 43 of RT. Seventeen percent (34/200) of patients were given cetuximab or nimotuzumab.

### 2.4 Follow‐up

Patients were followed every 3 months over the first 2 years, every 6 months over the following 3 years, and then annually thereafter. The last follow‐up was in August 2018. RT‐related toxicities were graded according to the RTOG radiation morbidity scoring criteria at each follow‐up.^10^

### 2.5 Statistical analysis

All data were analyzed using SPSS software version 19.0 (SPSS, Chicago, Illinois). All events were measured from the start of treatment, and the following end points (time to the first defined event) were assessed: overall survival (OS), disease‐free survival (DFS), local relapse‐free survival (LRFS), regional relapse‐free survival (RRFS), locoregional relapse‐free survival (LRRFS), and distant metastasis‐free survival (DMFS). The estimation was performed using the Kaplan‐Meier method. Multivariate analysis of significant factors was conducted using the Cox proportional hazard model. The comparison of dosimetric parameters in patients with and without recurrent disease was performed using the Mann‐Whitney *U* test. The relationship between dose and TLI was tested by using a logistic regression model. Receiver operating characteristic (ROC) curve analysis was used to evaluate different cutoff points for GTVnx volume and the maximal dose (Dmax) of the temporal lobe. Two‐sided tests were performed, and differences with *P* < .05 were considered statistically significant.

## 3 RESULTS

### 3.1 Treatment outcomes

The median follow‐up duration for all patients was 71 months (range, 8‐104 months). The 5‐year OS, DFS, LRFS, RRFS, LRRFS, and DMFS were 83.0%, 70.5%, 90.1%, 97.7%, 89.0%, and 82.4%, respectively. A total of 49 (24.5%) patients experienced treatment failures. Nineteen (9.5%) patients developed locoregional recurrences, and 32 (16.0%) patients developed distant metastases. Thirteen, 2, and 30 patients developed local failure, regional failure, and distant metastasis only, respectively. Two patients developed local and regional failure, 1 patient developed distant metastasis and failure at the primary site, and 1 patient had developed distant metastasis and failure at the nodal site. The median time to locoregional recurrence was 25.5 (range, 8‐73) months and 32.5 (range, 5‐60) months to distant metastases. In the study, 42 (21.0%) patients died during the follow‐up period. Thirty‐five died from tumor‐related causes, and 4 died from other causes, including 1 of profuse epistaxis, 1 of cardiac disease, and 2 of severe pneumonia, and 3 died from unknown causes.

### 3.2 Treatment‐related late toxicities

All patients had records of toxicity evaluation with more than 12 months of follow‐up were analyzed. Common late toxicities are listed in Table 2. The most common radiation‐related complication was xerostomia. A total of 156 (78.0%) patients had xerostomia at 12 months after RT. Other toxicities included ototoxicity in 44 (22.0%) patients, cranial nerve palsy in 4 (2.4%) patients, and optic nerve disorder in 5 patients (2.5%). Of 166 evaluable patients, MRI‐detected radiation‐induced TLI was identified in 17 (10.2%) patients, 3 with T3 disease, and 14 with T4 disease. Of them, 14 (82.4%) patients were asymptomatic, and 3 (17.6%) patients had clinical symptoms including vertigo, headaches, memory deterioration, and epileptic attacks. Twelve (70.6%) and 5 (29.4%) patients had radiation‐induced injuries to the unilateral and bilateral‐temporal lobes, respectively. The median latency of TLI was 43.5 months (range, 15‐83 months). No incidence of brainstem or spinal cord injury was observed at the last follow‐up visit.

**Table 2:** Major late toxicities in patients with T3‐4 nasopharyngeal carcinoma

Late toxicity	No. of patients (%) by grade 0	No. of patients (%) by grade 1	No. of patients (%) by grade 2	No. of patients (%) by grade 3	No. of patients (%) by grade 4
Temporal lobe injury	149 (89.8)	13 (7.8)	3 (1.8)	0	1 (0.6)
Optic nerve disorder	195 (97.5)	3 (1.5)	0	1 (0.5)	1 (0.5)
Ototoxicity	156 (78.0)	23 (11.5)	16 (8.0)	3 (1.5)	2 (1.0)
Xerostomia	44 (22.0)	133 (66.5)	20 (10.0)	3 (1.5)	0
Spinal cord injury	200 (100)	0	0	0	0
Brainstem injury	200 (100)	0	0	0	0

### 3.3 Prognostic factors

**Figure 1:**
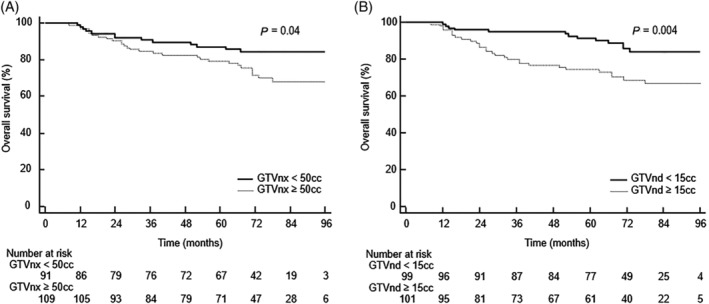
A, The Kaplan–Meier survival curves for patients with GTVnx volume <50 cc and ≥50 cc. B, The Kaplan–Meier survival curves for patients with GTVnd volume <15 cc and ≥15 cc. GTVnx, gross tumor volume including the primary nasopharyngeal tumor

GTVnx and GTVnd volume were significant prognostic factors in survival analysis (Figure 1A and B). The 5‐year OS rate for patients with GTVnx <50 cc and GTVnx ≥50 cc were 87.1% and 79.5%, respectively (log‐rank, *P* = .04), and the 5‐year OS rate for patients with GTVnd <15 cc and GTVnd ≥15 cc were 91.4% and 74.5%, respectively (log‐rank, *P* = .004).

Univariate analysis (Table S1) revealed that T4, clinical stage IVA/IVB, GTVnx ≥50 cc, and GTVnd ≥15 cc were related to a poor prognosis for OS and DFS and that GTVnd ≥15 cc was associated with poor DMFS but not LRFS or RRFS. However, multivariate analysis (Table 3) revealed that only GTVnd remained an independent prognostic factor for OS, DFS, and DMFS.

**Table 3:** Multivariate analysis of various factors on survivals of T3‐4 nasopharyngeal carcinoma (n = 200)

Characteristics	5‐year OS		5‐year DFS		5‐year DMFS	
	HR (95% CI)	*P*	HR (95% CI)	*P*	HR (95% CI)	*P*
T stage						
T3 vs.T4	0.797 (0.177‐3.584)	.77	1.216 (0.282‐5.250)	.79	1.540 (0.195‐12.127)	.68
Clinical stage						
III vs. IVA/IVB	2.608 (0.546‐12.467)	.23	1.182 (0.268‐5.210)	.82	0.941 (0.117‐7.574)	.96
Volume of GTVnx						
<50 cc vs. ≥50 cc	1.408 (0.693‐2.858)	.34	1.570 (0.877‐2.812)	.13	1.234 (0.558‐2.726)	.60
Volume of GTVnd						
<15 cc vs. ≥15 cc	2.316 (1.177‐4.560)	.02	2.705 (1.542‐4.747)	.001	3.359 (1.481‐7.619)	.004

Abbreviations: CI, confidence interval; DFS, disease‐free survival; DMFS, distant metastasis‐free survival; HR, hazard ratio; OS, overall survival; Volume of GTVnd, the sum volume of all involved lymph nodes; volume of GTVnx, gross tumor volume of nasopharynx.

### 3.4 Dosimetric analysis of local control

Dosimetric data for targets are shown in Table S2. The median D95 of the PGTVnx was 70.0 Gy (59.3‐75.8 Gy), and 51.5% (103/200) of patients were inadequately covered by a 95% prescribed dose of 70 Gy. In these 103 patients, the median D95 of the PGTVnx was 68.9 Gy (59.3‐70.0 Gy), and 5 patients with T4 stage received less than 66 Gy due to tumor closely abutting neurological structures.

The dosimetric parameters for patients with and without local relapse are summarized in Table 4. According to the dosimetric analysis of 200 patients with advanced T‐stage NPC treated with IMRT, the rate of local relapse and received dose to the PGTVnx, PCTV1, and PCTV2 were not significantly different.

**Table 4:** Dosimetric parameters for patients with and without local relapse (n = 200)

Parameters	Patients without local relapse (n = 181)		Patients with local relapse (n = 19)		*P*‐value
	Median	Range	Median	Range	
PGTVnx					
Volume (cc)	90.6	11.7‐257.4	76.7	25.7‐208.3	.40
D2 (Gy)	79.9	73.2‐85.7	81.3	75.2‐85.7	.22
D50 (Gy)	74.9	67.8‐79.6	76.8	71.3‐78.9	.34
D95 (Gy)	70.0	61.6‐75.8	70.0	59.3‐73.4	.81
D98 (Gy)	68.4	58.3‐74.8	69.0	54.7‐71.8	.78
PCTV1					
Volume (cc)	189.5	50.1‐850.8	158.8	80.34‐892.2	.32
D2 (Gy)	79.0	64.4‐84.6	79.7	66.0‐85.3	.27
D50 (Gy)	71.8	57.1‐78.6	72.6	64.2‐77.6	.56
D95 (Gy)	63.0	54.2‐74.6	63.4	55.1‐68.2	.72
D98 (Gy)	60.6	51.3‐73.4	61.1	52.1‐70.0	.57
PCTV2					
Volume (cc)	702.2	430.9‐1268.2	636.4	465.4‐1430.0	.43
D2 (Gy)	76.1	56.3‐82.4	76.6	58.1‐83.7	.48
D50 (Gy)	62.7	53.0‐72.9	63.4	55.6‐70.6	.62
D95 (Gy)	55.3	45.4‐68.5	55.0	50.0‐57.4	.42
D98 (Gy)	53.2	43.1‐68.0	52.8	46.7‐55.5	.37

Abbreviations: D2‐98, minimum dose to 2–98% volume; Volume, Volume of PGTVnx, PCTV1, PCTV2.

### 3.5 Dosimetric data to OAR with side effects

**Figure 2:**
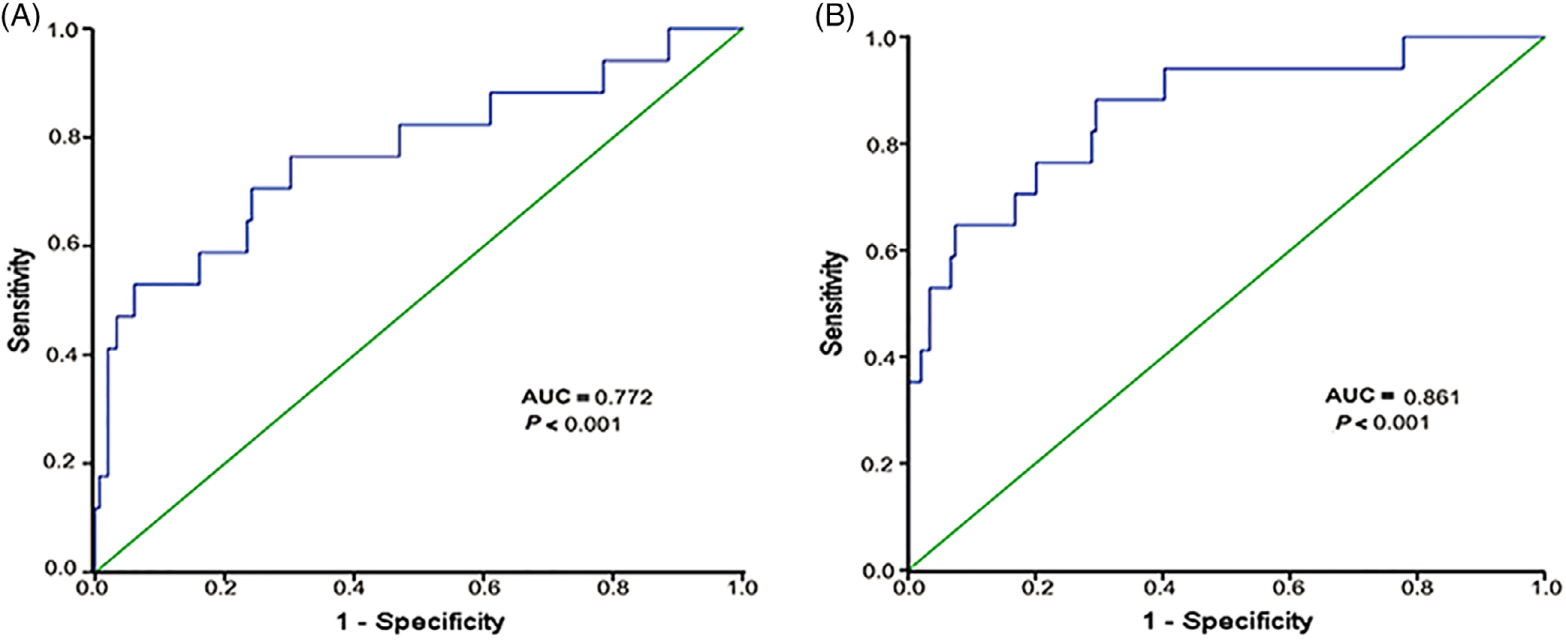
Receiver operating characteristic (ROC) curve for GTVnx volume (A) and the Dmax (B) to temporal lobe injury. GTVnx, gross tumor volume including the primary nasopharyngeal tumor [Color figure can be viewed at http://wileyonlinelibrary.com]

Dosimetric data for OARs are shown in Table S3. The maximum median dose to the brainstem was 59.6 Gy (39.2‐75.3 Gy), and approximately 79.5% (159/200) patients had a maximum dose to the brainstem exceeding 54 Gy. The maximum median point doses to the spinal cord and optic chiasm were 39.6 Gy (29.9‐59.0 Gy) and 57.1 Gy (6.9‐81.3 Gy), respectively. Only 2 patients received maximum dose less than 60 Gy to the temporal lobe. The maximum median dose to the left temporal lobe and right temporal lobe was 76.5 Gy (59.2‐88.2 Gy) and 73.7 Gy (59.3‐89.4 Gy), respectively. The temporal lobes exceeded the limiting dose mainly because of extension of the tumor to the cavernous sinus, parasellar region, or posterior cranial fossa. Of the 166 evaluable patients after IMRT, 17 had TLI. According to the logistic analysis of dosimetric factors in TLI, the odds ratio attributed to the volume of the primary tumor to the TLI was 1.035 (95% confidence interval (CI), 1.006‐1.065; *P* = .02), and the Dmax was 1.006 (95% CI, 1.002‐1.009; *P* = .004). ROC analysis was used to evaluate different cutoff points for the GTVnx volume and Dmax to the TLI. The cutoff point for the GTVnx volume was 93 cc (sensitivity 52.9%, specificity 94%; area under the ROC curve [AUC] = 0.772, *P* < .001, Figure 2A). The cutoff point for Dmax was 78 Gy (sensitivity 88.2%, specificity 70.5%; AUC = 0.861, *P* < .001, Figure 2B).

## 4 DISCUSSION

IMRT is an advanced form of conformal RT that permits more precise cancer targeting while reducing the dose to normal tissues.^11^ IMRT provides satisfactory long‐term outcomes for patients with NPC, with acceptable late toxicities.^12^ IMRT is an effective radiation treatment that achieves a high rate of locoregional control of approximately 80%‐90% after comprehensive treatment.^13^, ^14^ Distant metastases become the main pattern of treatment failure, particularly in patients with advanced disease. In a randomized study by Peng,^15^ IMRT contributed to an absolute improvement in the 5‐year locoregional control of 7.7% and a 5‐year OS of 12.5% compared with conventional 2D conventional radiation therapy (2D‐CRT), similar to results reported by Lee.^16^ Au et al^12^ retrospectively reviewed the data of 3328 patients with NPC treated with IMRT or 2D‐CRT and found that IMRT provided a higher local tumor control rate with a lower incidence of late complications. The 5‐year local control rate for T1‐3 disease exceeded 90%, but the local control rate of T4 was 71.6%, as T4 often lies close to nearby critical neurological structures, compromising the radiation dose coverage of the tumor volume and therefore undermining local control. Ou et al^17^ performed a retrospective analysis of 869 patients with nonmetastatic NPC and reported a 5‐year local control rate of 89.7% and a 5‐year OS rate of 84.0%. Multivariate analysis showed that age, T‐stage, N‐stage, and the total dose of cisplatin were independent prognostic factors for OS. Our results, with a median follow‐up duration of more than 5 years, showed that the 5‐year OS, DFS, LRRFS, and DMFS rates were 83.0%, 70.5%, 89.0%, and 82.4%, respectively. The outcomes of patients with stage III and stage IVA/IVB NPC were highly satisfactory.

In previous studies, T‐stage and primary tumor volume had significant impact on the prognosis of patients with NPC. Chen et al^18^ reported that the 5‐year OS rate was significantly reduced for patients with a large tumor volume (> 50 mL). Ng et al^19^ reported that 48 cm^3^ was identified as the critical cutoff GTV_P volume, and the large volume group (GTV_P ≥ 48 cm^3^) had poorer 5‐year DFS and OS rates than the small volume group. Similar results were also found in our study.

In addition, although the N classification was confirmed to be one of the most relevant factors in the prognosis of NPC, it is still unclear whether the GTVnd affects the prognosis. Here, we demonstrated that the GTVnd was identified as an independent prognostic factor for OS, DFS, and DMFS. Patients with a GTVnd volume ≥15 cc had a high risk of distant metastasis and poorer treatment outcomes.

Dosimetric inadequacy of the primary tumor volume may lead to recurrence, while high doses received by normal tissues may cause serious complications. In advanced NPC, radiation injury to important organs adjacent to the tumor, such as the brainstem, spinal cord, optical nerve, and temporal lobe will seriously affect the quality of life in patients. Therefore, in treatment planning, they are class I priority organs with weights higher than that of the target. Radiation field coverage and dose are often compromised by limitations imposed by the proximity of the tumor to these critical organs. The aim of treatment for locally advanced NPC is to improve local control by escalating the radiation dose to the tumor without exceeding the tolerance of normal tissue. Ng et al^19^ found that an underdosed GTVp of 3.4 cm^3^ was a prognostic factor for local failure. The local control rate tends to increase with the escalation in radiation dose, and planning needs to balance the dose requirements between targets and OARs.

Table 4 compares various dosimetric parameters in patients with and without local recurrences, which did not show statistically significant differences. We observed that for patients with T4 NPC, especially those with tumors close to the brainstem and other important OARs, slight dosimetric inadequacy did not lead to an increase in tumor recurrence; furthermore, when the tumor invaded close to the brainstem and other important OARs and the organ‐defined dose could not be achieved, a certain degree of exceeding dose to normal tissues did not increase late toxicities of the central nervous system.

Despite the advantages of IMRT, radiation‐induced late toxicities remain the treatment bottleneck for locally advanced NPC. Assessing quality of life for patients with NPC has become an important issue. Xerostomia is a common long‐term side effect of RT, which can be reduced with the use of IMRT.^20^, ^21^ The incidence of radiation‐induced TLI was 1.9%‐13.1% after IMRT,^15^, ^22^ which most likely occurs in patients with advanced T‐stage disease. In this study, the incidence of TLI in patients with T3‐4 stage was 10.2%. The differences in treatment strategy, temporal lobe delineation, and follow‐up period might cause variation in the incidence. In 2010, Lawrence reported that the irradiation dose, fractionated dose, and irradiation volume were important factors affecting the occurrence of temporal lobe necrosis.^23^Sun et al^24^ reported that risk of TLI was highly dependent on the high dose to the temporal lobe. They used 0.5 cc of temporal lobe volume that received 69 Gy as a cutoff. Cheng et al^25^ reported that the crude TLI rate after IMRT was 12.9%; they used the least absolute shrinkage and selection operator method to select Dmax and D1cc, which were the most significant predictors of TLI development. A study involving 749 patients with NPC demonstrated that the risk of TLI increased at D0.5cc ≥ 73.66Gy.^26^ In a retrospective study of T4 NPC, D1cc ≤ 71.14 Gy was helpful in reducing the incidence of TLI.^27^ In our study, there were 14 patients at T4 stage (82.4%) with TLI. TLI occurred in the field receiving the highest dose, which was next to the GTVnx. The dosimetric analysis showed that patients with TLI were exposed to high radiation doses and that the Dmax of the temporal lobe was a predictor of TLI consistent with previous studies. In Quantitative Analysis of Normal Tissue Effects in the Clinic study,^28^ a dose‐response relationship was found in the brain. The incidence of radio necrosis increased from 3% with Dmax <60 Gy to 5% with Dmax of 72 Gy, and to 10% with Dmax of 90 Gy in the 2 Gy per fraction. This result suggests that a small volume of the brain can tolerate higher doses of radiation therapy. Therefore, it is usually necessary to relax the dose limit on brain tissue during treatment planning to satisfy the dose coverage of the target. Because NPC is highly curative, and the radiation‐induced brain injury mainly locates in the temporal lobe, frontal lobe, and other nonfunctional areas which have less effect on quality of life, the dose limit on brain tissue should be loosened to allow a small volume of brain to receive a higher dose of radiation.

## 5 CONCLUSIONS

With the application of IMRT in NPC, the long‐term outcomes have been remarkable, showing locoregional control of approximate 90% at 5 years, and distant metastasis becomes the most common pattern of treatment failure in patients with T3‐4 disease. Treatment‐related toxicities are well tolerable. We demonstrated that the GTVnd is an independent prognostic factor. The dose constraint criteria of selected critical neurological structures can be appropriately loosened without increasing the rate of severe late toxicities. The significant factors affecting the risk of TLI included GTVnx volume and Dmax. Our results suggest that restricting Dmax <78 Gy could help to significantly reduce the occurrence of TLI in advanced T‐stage NPC. However, the dosimetric parameters between tumors and OARs require further investigation to improve tumor control and avoid side effects.

## CONFLICT OF INTEREST

The authors declare no potential conflict of interest.

## Supporting information


**Table S1**: Univariate analysis of various factors on survivals of T3‐4 nasopharyngeal carcinoma (n = 200).

**Table S2**: Dose‐volume histogram statistics of targets (n = 200).

**Table S3**: Dosimetric data for neurological organs at risk (n = 200).
