# Supplementary material for: The relations of dosimetric parameters with long‐term outcomes and late toxicities in advanced T‐stage nasopharyngeal carcinoma with IMRT
Source: Head Neck. 2019 Oct 24;42(1):85–92. doi: 10.1002/hed.25986 (PMC6973082; doi:10.1002/hed.25986)
Supplement: Supplementary file 1 — Table S1: Univariate analysis of various factors on survivals of T3‐4 nasopharyngeal carcinoma (n = 200). [file HED-42-85-s001.docx]

Supplementary Table 1

Univariate analysis of various factors on survivals of T3-4 nasopharyngeal carcinoma (n = 200).

| Characteristics | Number | 5y-OS (%) | *p* | 5y-DFS (%) | *p* | 5y-LRFS (%) | *p* | 5y-RRFS (%) | *p* | 5y-DMFS (%) | *p* |
| --- | --- | --- | --- | --- | --- | --- | --- | --- | --- | --- | --- |
| Gender |  |  |  |  |  |  |  |  |  |  |  |
| Male | 147 | 78.9 | 0.959 | 68.0 | 0.620 | 91.2 | 0.598 | 97.3 | 0.225 | 83.7 | 0.834 |
| Female | 53 | 79.2 |  | 71.7 |  | 88.7 |  | 100 |  | 84.9 |  |
| Age |  |  |  |  |  |  |  |  |  |  |  |
| < 46 | 91 | 81.3 | 0.462 | 71.4 | 0.497 | 94.5 | 0.078 | 98.9 | 0.406 | 83.5 | 0.865 |
| ≥ 46 | 109 | 77.1 |  | 67.0 |  | 87.2 |  | 97.2 |  | 84.4 |  |
| T stage |  |  |  |  |  |  |  |  |  |  |  |
| T3 | 80 | 86.3 | 0.040 | 76.3 | 0.070 | 91.3 | 0.768 | 98.8 | 0.536 | 87.5 | 0.270 |
| T4 | 120 | 74.2 |  | 64.2 |  | 90.0 |  | 97.5 |  | 81.7 |  |
| N stage |  |  |  |  |  |  |  |  |  |  |  |
| N0-1 | 77 | 79.2 | 0.952 | 72.7 | 0.367 | 90.9 | 0.876 | 97.4 | 0.633 | 84.4 | 0.899 |
| N2-3 | 123 | 78.9 |  | 66.7 |  | 90.2 |  | 98.4 |  | 83.7 |  |
| Clinical stage |  |  |  |  |  |  |  |  |  |  |  |
| Ⅲ | 74 | 87.8 | 0.019 | 77.0 | 0.060 | 90.5 | 0.988 | 98.6 | 0.616 | 87.8 | 0.257 |
| ⅣA/ⅣB | 126 | 73.8 |  | 64.3 |  | 90.5 |  | 97.6 |  | 81.7 |  |
| Chemotherapy |  |  |  |  |  |  |  |  |  |  |  |
| ≤ 4 cycles | 102 | 80.4 | 0.622 | 71.6 | 0.423 | 90.2 | 0.881 | 96.1 | 0.048 | 88.2 | 0.096 |
| > 4 cycles | 98 | 77.6 |  | 66.3 |  | 90.8 |  | 100 |  | 79.6 |  |
| Volume of GTVnx | |  |  |  |  |  |  |  |  |  |  |
| < 50cc | 91 | 85.7 | 0.033 | 79.1 | 0.006 | 89.0 | 0.512 | 98.9 | 0.406 | 87.9 | 0.168 |
| ≥ 50cc | 109 | 73.4 |  | 60.6 |  | 91.7 |  | 97.2 |  | 80.7 |  |
| Volume of GTVnd | |  |  |  |  |  |  |  |  |  |  |
| < 15cc | 99 | 86.9 | 0.007 | 81.8 | 0.000 | 91.9 | 0.498 | 99.0 | 0.322 | 91.9 | 0.002 |
| ≥ 15cc | 101 | 71.3 |  | 56.4 |  | 89.1 |  | 97.0 |  | 76.2 |  |

Abbreviation: Volume of GTVnx = gross tumor volume of nasopharynx; Volume of GTVnd = the sum volume of all involved lymph nodes; OS = overall survival; DFS = disease-free survival; LRFS = local relapse-free survival; RRFS = regional relapse-free survival; DMFS = distant metastasis-free survival.
