# Supplementary material for: The relations of dosimetric parameters with long‐term outcomes and late toxicities in advanced T‐stage nasopharyngeal carcinoma with IMRT
Source: Head Neck. 2019 Oct 24;42(1):85–92. doi: 10.1002/hed.25986 (PMC6973082; doi:10.1002/hed.25986)
Supplement: Supplementary file 2 — Table S2: Dose‐volume histogram statistics of targets (n = 200). [file HED-42-85-s002.docx]

Supplementary Table 2

Dose-volume histogram statistics of targets (n = 200).

| Target | Parameters | Dosimetric data | Median difference of insufficient prescription dose (Gy) | Proportion of insufficient prescription dose (%) |
| --- | --- | --- | --- | --- |
| PGTVnx | Volume(cc) | 89.6 (11.7-257.4) | - | - |
|  | D2(Gy) | 80.0 (73.2-85.7) | - | - |
|  | D50(Gy) | 75.1 (67.8-79.6) | - | - |
|  | D95(Gy) | 70.0 (59.3-75.8) | 0 (-10.7-5.8) | 51.5 (103/200) |
|  | D98(Gy) | 68.5 (54.7-74.8) | - | - |
|  | CI | 0.43 (0.04-0.76) | - | - |
|  | HI | 0.15 (0.07-0.32) | - | - |
| PGTVnd | Volume(cc) | 34.4 (0.3-216.0) | - | - |
|  | D2(Gy) | 76.2 (63.9-82.3) | - | - |
|  | D50(Gy) | 72.9 (60.0-78.3) | - | - |
|  | D95(Gy) | 69.5 (55.5-74.9) | -0.5 (-14.5-4.9) | 72.0 (144/200) |
|  | D98(Gy) | 68.1 (53.5-74.5) | - | - |
|  | CI | 0.16 (0.29-0.47) | - | - |
|  | HI | 0.11 (0.04-0.76) | - | - |
| PCTV1 | Volume(cc) | 187.6 (50.0-892.2) | - | - |
|  | D2(Gy) | 79.1 (64.4-85.3) | - | - |
|  | D50(Gy) | 71.8 (57.1-78.6) | - | - |
|  | D95(Gy) | 63.0 (54.2-74.6) | 3.0 (-5.8-14.6) | 23.0 (46/200) |
|  | D98(Gy) | 60.6 (51.3-73.4) | - | - |
|  | CI | 0.33 (0.11-0.79) | - | - |
|  | HI | 0.24 (0.04-0.45) | - | - |
| PCTV2 | Volume(cc) | 697.0(430.9-1430.0) | - | - |
|  | D2(Gy) | 76.2(56.3-83.7) | - | - |
|  | D50(Gy) | 63.1(53.0-72.9) | - | - |
|  | D95(Gy) | 55.2(45.4-68.5) | 5.2 (-4.6-18.5) | 2.0 (4/200) |
|  | D98(Gy) | 53.2(43.1-68.0) | - | - |
|  | CI | 0.53(0.11-0.80) | - | - |
|  | HI | 0.35(0.07-0.48) | - | - |

All values are presented as median followed by range in parenteheses

Abbreviation: PGTVnx = primary nasopharyngeal tumor; PGTVnd = involved lymph nodes; PCTV1 = planning target volumes GTVnx plus the whole nasopharynx; PCTV2 = PTV1 with low-risk local structures and lymphatic regions; D2 = dose to 2% volume; D50= dose to 50% volume; D95 = dose to 95% volume; D98 = dose to 98% volume; CI = conformity index; HI = homogeneity index.
