# Supplementary material for: The relations of dosimetric parameters with long‐term outcomes and late toxicities in advanced T‐stage nasopharyngeal carcinoma with IMRT
Source: Head Neck. 2019 Oct 24;42(1):85–92. doi: 10.1002/hed.25986 (PMC6973082; doi:10.1002/hed.25986)
Supplement: Supplementary file 3 — Table S3: Dosimetric data for neurological organs at risk (n = 200). [file HED-42-85-s003.docx]

Supplementary Table 3

Dosimetric data for neurological organs at risk (n = 200).

| Organ at risk | Parameters | Dosimetric data (Gy) | Median difference of exceed the limit dose (Gy) | Proportion of exceed the limit dose (%) |
| --- | --- | --- | --- | --- |
| Brainstem | Dmax | 59.6 (39.2-75.3) | 5.6 (-14.8-21.3) | 79.5 (159/200) |
|  | Dmean | 30.3 (16.0-44.4) |  |  |
| Spinal cord | Dmax | 39.6 (29.9-59.0) | -0.4 (-10.1-19.0) | 44.5 (89/200) |
|  | Dmean | 22.3 (7.3-32.4) |  |  |
| Left temporal lobe | Dmax | 76.5 (59.2-88.2) | 16.5 (-0.8-28.2) | 99 (198/200) |
|  | Dmean | 22.4 (10.2-52.3) |  |  |
| Right temporal lobe | Dmax | 73.7 (59.3-89.4) | 13.7 (-0.7-29.4) | 99 (198/200) |
|  | Dmean | 22.2 (8.9-44.3) |  |  |
| Optic chiasm | Dmax | 57.1 (6.9-81.3) | 3.1 (-47.0-27.3) | 63.5 (127/200) |
|  | Dmean | 45.5 (4.5-74.0) |  |  |
| Left optic nerve | Dmax | 63.7 (7.2-86.6) | 9.6 (-46.8-32.6) | 80.5 (161/200) |
|  | Dmean | 33.8 (6.5-74.6) |  |  |
| Right optic nerve | Dmax | 64.8 (6.0-88.8) | 10.6 (-48.0-34.8) | 78.0 (156/200) |
|  | Dmean | 36.3 (4.9-66.0) |  |  |

All values are presented as median followed by range in parenteheses.

Limit dose of brainstem, spinal cord, temporal lobe, optic chiasm, optic nerve were 54Gy, 40Gy, 60Gy, 54Gy, 54Gy, respectively.

Abbreviation: Dmax = maximum dose; Dmean= mean dose.
